# K63-linked ubiquitination of DYRK1A by TRAF2 alleviates Sprouty 2-mediated degradation of EGFR

**DOI:** 10.1038/s41419-021-03887-2

**Published:** 2021-06-11

**Authors:** Pengshan Zhang, Zhe Zhang, Yinkun Fu, Ying Zhang, Michael P. Washburn, Laurence Florens, Min Wu, Chen Huang, Zhaoyuan Hou, Man Mohan

**Affiliations:** 1 Tongren Hospital/Faculty of Basic Medicine, Hongqiao Institute of Medicine, Shanghai Jiaotong University School of Medicine, Shanghai, China; 2 Shanghai Key Laboratory of Tumor Microenvironment and Inflammation, Department of Biochemistry and Molecular Cell Biology, Shanghai Jiaotong University School of Medicine, Shanghai, China; 3 Department of General Surgery, Shanghai General Hospital, Shanghai Jiaotong University School of Medicine, Shanghai, China; 4 State Key Laboratory of Primate Biomedical Research, Institute of Primate Translational Medicine, Kunming University of Science and Technology, Kunming, Yunnan China; 5 Stowers Institute for Medical Research, Kansas City, MI USA; 6 Department of Pathology and Laboratory Medicine, The University of Kansas Medical Center, Kansas City, KS USA; 7 Hubei Key Laboratory of Cell Homeostasis, College of Life Sciences, Wuhan University, Wuhan, Hubei China

**Keywords:** Kinases, Ubiquitins, Ubiquitylation, Cancer in the nervous system

## Abstract

Dual specificity tyrosine phosphorylation regulated kinase 1A, DYRK1A, functions in multiple cellular pathways, including signaling, endocytosis, synaptic transmission, and transcription. Alterations in dosage of DYRK1A leads to defects in neurogenesis, cell growth, and differentiation, and may increase the risk of certain cancers. DYRK1A localizes to a number of subcellular structures including vesicles where it is known to phosphorylate a number of proteins and regulate vesicle biology. However, the mechanism by which it translocates to vesicles is poorly understood. Here we report the discovery of TRAF2, an E3 ligase, as an interaction partner of DYRK1A. Our data suggest that TRAF2 binds to PVQE motif residing in between the PEST and histidine repeat domain (HRD) of DYRK1A protein, and mediates K63-linked ubiquitination of DYRK1A. This results in translocation of DYRK1A to the vesicle membrane. DYRK1A increases phosphorylation of Sprouty 2 on vesicles, leading to the inhibition of EGFR degradation, and depletion of TRAF2 expression accelerates EGFR degradation. Further, silencing of DYRK1A inhibits the growth of glioma cells mediated by TRAF2. Collectively, these findings suggest that the axis of TRAF2–DYRK1A-Sprouty 2 can be a target for new therapeutic development for EGFR-mediated human pathologies.

## Introduction

Dual specificity tyrosine phosphorylation regulated kinase 1A (DYRK1A) is a member of the CMGC (including cyclin-dependent kinases (CDKs), mitogen-activated protein kinases (MAP kinases), glycogen synthase kinases (GSK) and CDK-like kinases) group of serine/threonine kinases [1]. Owing to its localization within the Down Syndrome Critical Region on chromosome 21, and due to its dosage-sensitive functions, it is believed to contribute to Down syndrome phenotypes [2]. DYRK1A is implicated in embryonic development and cellular growth, and it regulates the balance between proliferation and differentiation in neural progenitors, in developing nervous system [2, 3]. DYRK1A’s function is dosage sensitive and alterations in its expression levels lead to pathology [4, 5]. A number of heterozygous mutations in DYRK1A in or near the kinase domain are associated with DYRK1A syndrome, which exhibit growth retardation, microcephaly, and intellectual disability [4, 5]. Similar phenotypes were observed in heterozygous DYRK1A mice [6, 7]. Growth retardation and a small brain phenotype is also observed due to loss of DYRK1A homologs in *Drosophila* and zebrafish [8, 9]. Reduced brain size in DYRK1A loss-of-function models is caused by a reduction in the number of neurons in some parts of the brain, whereas other areas see an increased number of neurons [10]. DYRK1A is shown to phosphorylate several proteins involved in neuronal synaptic transmission, including Dynamin 1, Amphiphysin 1, and Synaptojanin 1 [2, 11–14], and functions in synaptic vesicle endocytosis [11]. Overall, these studies indicate a function of DYRK1A in neuronal development.

Down syndrome patients exhibit an increased incidence of leukemia and a three-copy Dyrk1a mouse model develops acute megakaryoblastic leukemia [15]. In the gliomas, high expression of DYRK1A correlates with epidermal growth factor receptor (EGFR) expression. DYRK1A prevents endocytotic degradation of EGFR through phosphorylation of the EGFR-signaling modulator Sprouty 2 (SPRY2) and DYRK1A inhibition leads to reduced glioma growth [16, 17]. These observations implicate DYRK1A in tumorigenesis.

Over the past two decades, a large number of DYRK1A substrates located in various subcellular structures, including the nucleus, cytoplasm, cytoskeleton, and vesicles, have been identified, suggesting a wide variety of cellular functions for DYRK1A [18]. A number of studies have shown that DYRK1A associates with and phosphorylates multiple proteins found in the vesicles, and affects the synaptic vesicle, EGFR, and Transferrin endocytosis [2]. The regulation of DYRK1A localization and function in the vesicles, however, is not clear.

Tumor necrosis factor receptor (TNFR)-associated factor 2 (TRAF2) is an adaptor protein in the TNF-induced signaling pathway. Upon ligand binding to TNFR1 and TNFR2, TRAF2 is recruited to the plasma membrane and promotes the activation of canonical nuclear factor-κB (NF-κB) pathway and JNK/p38 pathways [19]. TRAF2 harbors a RING domain, which primarily mediates K63-linked ubiquitination of substrates [20–23], and a TRAF domain, which has scaffolding activity [24]. Recent reports indicate that TRAF2 is also involved in other pathways, including activation of NF-κB pathway induced by nucleotide-binding oligomerization domain-like receptors, in a RIG-I-like receptor-mediated antiviral response pathway and cytokine receptor signaling [25, 26]. Further, some recent studies have reported TRAF2 to be upregulated in multiple cancer types including the glioma and can serve as prognostic biomarker [27–29].

In this study, we have identified TRAF2 as an interaction partner of DYRK1A. We found that TRAF2 mediates K63-linked ubiquitination of DYRK1A and causes its translocation into vesicles, where DYRK1A interacts with and phosphorylates SPRY2. This TRAF2–DYRK1A–SPRY2 axis regulates the stability of EGFR, which could be significant in the EGFR-dependent glioblastoma or other cancers.

## Materials and methods

### Plasmids

The human TRAF2 cDNA was subcloned into a pCMV vector with an N-terminal Myc tag to generate Myc-TRAF2. The mouse Traf2 cDNA was subcloned into pCDH-CMV lentiviral vector with an N-terminal hemagglutinin (HA) tag to generate HA-Traf2. pCDNA5-Flag-Dyrk1a plasmid has been previously described [30]. Dyrk1a cDNA was also subcloned into a doxycycline-inducible expression lentiviral vector pLUT (kindly gifted by Dr. Zhaoyuan Hou from Shanghai Jiaotong University School of Medicine, Shanghai, China) with an N-terminal Flag tag. pIP-Flag-ub-K48-only and pIP-Flag-ub-K63-only plasmids were kindly gifted by Dr. Xuemei Tong (Shanghai Jiaotong University School of Medicine, Shanghai, China), and we replaced the Flag tag with Myc tag. All Myc-TRAF2 truncations, Flag-Dyrk1a mutants, and Myc-ub mutants were made by using site-directed mutagenesis procedures following the manufacturer’s protocol (Toyobo, Japan). The pLX304-SPRY2-V5 expression plasmid (cDNA BC015745.1) was obtained from Thermo Fisher. All the constructs were confirmed by DNA sequencing. Lentiviral vectors to silence human DYRK1A has been previously described [30]. Short hairpin RNA (shRNA) targeting human TRAF2 or mouse Dyrk1a were from GenePharma Co., Ltd (Shanghai, China). The sequences to silence human DYRK1A were shDYRK1A-1, 5′-GGTATTCCACCTGCTCATA-3′ and shDYRK1A-2, 5′-TGACAGGAGTTTGTGTGCA-3′. The sequence to silence mouse Dyrk1a was shDyrk1a, 5′-TGACTACTTGAAGTTCAAA-3′. The sequences to silence human TRAF2 were shTRAF2-1, 5′-CGACGTGACTTCATCCTCT-3′ and shTRAF2-2, 5′-TGGACCAAGACAAGATTGA-3′.

### Cell culture, viral production, and infection

HEK293, HEK293T, SH-SY5Y, NIH3T3, U251, and A172 cells were obtained from the American Type Culture Collection. All cell lines were cultured in Dulbecco’s modified Eagle’s medium supplemented with 10% fetal bovine serum (Gibco), 50 U/ml penicillin, and 50 µg/ml streptomycin (Hyclone). All cell lines were maintained at 37 °C under 5% CO_2_ in a humidified chamber. For viral production, lentiviral vectors and packaging plasmids (psPAX and pMD2.G plasmids) were transfected into HEK293T cells with polyethylenimine (PEI; 23966, Polysciences) or Lipofectamine 3000 (L3000-008, Invitrogen). Two days later, cell culture supernatant containing lentiviruses was collected and filtered through 0.45 μm filter. Cells were infected with the lentiviruses in the presence of 10 μg/ml polybrene (sc-134220, Santa Cruz) and selected with puromycin (60210ES25, Yeasen, China) for at least 4 days before functional analysis.

### Cell proliferation, colony formation assay, and wound-healing assay

For cell proliferation analysis, cells were plated at 100,000 cells per well of 6-well plates and then cell numbers were counted with trypan blue exclusion staining at day 1, 2, and 3 after plating. For colony formation, cells in single-cell suspension were plated into 6 cm dishes at a density of 500 cells for 14 days, then the growth medium was removed, and each dish was washed with phosphate-buffered saline (PBS), the colonies were fixed with 4% paraformaldehyde, and stained with 0.1% crystal violet. Excess crystal violet was washed off with double-distilled water. Cell images were captured and the numbers of cells were counted. Experiments were performed in triplicate. Wound-healing assays were performed as previously described [31].

### Separation of subcellular compartments

Cells in the dishes were rinsed twice with PBS and lysed in 0.5% Triton X-100 lysis buffer (0.5% Triton X-100, 20 mM HEPES pH 7.4, 150 mM NaCl, 2 mM EDTA) supplemented with protease and phosphatase inhibitors (Sigma, USA), and incubated on ice for 30 min. Lysates were centrifuged at 17,000 × *g*, 4 °C for 15 min, and supernatant taken as 0.5% Triton X-100 soluble fraction. The pellets were washed three times in 0.5% Triton X-100 lysis buffer and resuspended in 2% SDS lysis buffer (2% SDS, 50 mM Tris-HCl pH 6.8, 6% glycerol), heated at 95 °C for 10 min, and sonicated. Lysates were centrifuged at 17,000 × *g* for 15 min and the supernatant taken as 0.5% Triton X-100 insoluble fraction.

### Antibodies

Antibodies used in the study were as follows: anti-Flag (F3165/F7425, Sigma), anti-Myc (9B11/71D10, Cell Signaling Technology (CST)), anti-HA (16B12, Biolegend and H6908, Sigma), anti-TRAF2 (sc-136999, Santa Cruz), anti-Rab5 (sc-46692, Santa Cruz), anti-Rab7 (sc-376362, Santa Cruz), anti-Sprouty 2 (sc-100862, Santa Cruz/ab85670, Abcam), anti-EGFR (sc-373746, Santa Cruz), anti-p-Tyr (9411, CST), anti-p-Ser/Thr (ab9344, Abcam and 05-368, Sigma), anti-Ser (sc-81514, Santa Cruz), anti-Thr (sc-5267, Santa Cruz), anti-Ubiquitin (sc-8017, Santa Cruz), anti-β-Actin (AC026, Abclonal), anti-GFP (AE011, Abclonal), anti-glutathione *S*-transferase (GST) (2622 S, CST), anti-V5 (R960-25, Thermo Fisher and 30801ES10, Yeasen), anti-Mouse/Rabbit IgG-peroxidase secondary antibody (A0545/A9044, Sigma), anti-Mouse IgG (light chain specific)-peroxidase secondary antibody (115-005-174, Jackson), and anti-DYRK1A polyclonal antibody has been previously described [30].

### Immunoblotting

Cells in the dishes were rinsed twice with PBS, then cells were scraped into radioimmunoprecipitation assay lysis buffer (50 mM Tris-HCl pH 7.4, 150 mM NaCl, 1% Triton X-100, 0.25% sodium deoxycholate, 0.1% SDS, 1 mM EDTA supplemented with protease and phosphatase inhibitors) followed by 30 min on ice, or scraped into 2% SDS lysis buffer (2% SDS, 50 mM Tris-HCl pH 6.8, 6% glycerol) followed by heating at 95 °C for 10 min. Lysates were sonicated and centrifuged at 17,000 × *g*, 4 °C for 15 min. Protein concentration was measured with BCA Protein Assay Kit (23227, Thermo Fisher). Protein samples were separated on SDS-polyacrylamide gel electrophoresis (SDS-PAGE) and transferred onto nitrocellulose membranes. After blocking, blots were incubated with primary antibodies at 4 °C overnight, then incubated with secondary antibodies labeled with horseradish peroxidase at room temperature (RT) for 1 h and the signals were detected using the ECL Kit (WBKLS0100, Millipore). Western blot signals were quantified with Image J software.

### Co-immunoprecipitation and GST pulldown

Cells in the dishes were rinsed twice with PBS, then cells were scraped into IP lysis buffer (20 mM Tris-HCl pH 7.4, 150 mM NaCl, 0.5% Triton X-100, 2 mM EDTA) supplemented with phosphatase and protease inhibitors (Sigma, USA), and incubated on ice for 30 min. Lysates were centrifuged at 17,000 × *g*, 4 °C for 15 min, then the supernatant (soluble fraction) was reserved. The pellets were suspended in the same lysis buffer containing 60 mM *N*-octylglucoside (Sangon Biotech, China) on ice for 30 min. Extracts were spun down at 17,000 × *g*, 4 °C for 15 min and the supernatant (insoluble fraction) was reserved. Then soluble fraction and insoluble fraction were mixed and incubated with anti-Flag M2 affinity gel (A2220, Sigma) or anti-Myc agarose affinity gel (Sigma, USA) for 4 h at 4 °C, or incubated with protein A or protein A/G beads (Millipore, USA) and anti-TRAF2/anti-DYRK1A/anti-V5 antibody overnight at 4 °C. Then beads were washed four times and eluted with 2× SDS loading buffer. Samples were subjected to immunoblotting.

The GST pull-down assays were described previously [32]. GST-TRAF2 was expressed in *Escherichia coli* BL21 cells, which were induced by isopropyl-β-d-thiogalactoside (IPTG), and purified by Glutathione Sepharose beads (17-0756-01, GE Healthcare). For the in vitro binding assays, GST-TRAF2 protein was mixed with Flag-DYRK1A protein purified from HEK293T cells overnight.

### Large-scale Flag affinity purification and MudPIT analysis

Approximately 10^9^ cells for each cell line were collected and washed with PBS. Cells were swollen for 15 min in hypertonic buffer (Buffer A: 10 mM Hepes pH 7.9, 1.5 mM MgCl_2_, 10 mM KCl, 0.5 mM dithiothreitol) freshly supplemented with protease inhibitor cocktail (P8340, Sigma). Swollen cells were dounced 20 times in a Wheaton dounce homogenizer till about 90% cells were lysed (as observed in a microscope). Lysate is then centrifuged at 25,000 × *g* for 20 min, to pellet nuclei and cell debris. To the supernatant (S100), 0.11 volume of Buffer B (0.3 M Hepes pH 7.9, 1.4 M KCl, and 0.03 M MgCl_2_) was added and cleared by centrifuging at 100,000 × *g*, 4 °C for 1 h. The supernatant was treated as cytoplasmic extract and Flag-affinity was performed by incubating Flag beads for 3–4 h. Beads were then washed with Flag wash buffer (10 mM HEPES pH 7.9, 1.5 mM MgCl_2_, 300 mM NaCl, 10 mM KCl, 0.2% Triton X-100) three times and then eluted two times in elution buffer (200 μg/ml Flag peptide, 10 mM HEPES pH 7.9, 0.1 M NaCl, 1.5 mM MgCl_2_, 0.05% Triton X-100). Eluates were analyzed by silver staining and western blotting. Trichloroacetic acid-precipitated protein mixtures from the Flag purifications were digested with endoproteinase Lys-C and trypsin (Roche), and analyzed by a ten-step MudPIT (Multidimensional Protein Identification Technology) on an LTQ linear ion trap mass spectrometer (Thermo Fisher Scientific, USA) coupled to a Quaternary Agilent 1100 series high-performance liquid chromatography pump and a nano-liquid chromatography electrospray ionization source, as previously described [33].

### Mass spectrometry data processing and analysis

Tandem mass spectra were interpreted using ProluCID (10.1016/j.jprot.2015.07.001) against a database consisting of 44,093 non-redundant human proteins (collated from Genome Reference Consortium Human Build 38 patch release 13 and NCBI RefSeq 2019-12-03 release), 426 usual contaminants (human keratins, IgGs, and proteolytic enzymes), and the shuffled sequences of each non-redundant entry to estimate false discovery rates (FDRs). DTASelect (10.1021/pr015504q) and swallow, an in-house developed software (v. 0.0.1, https://github.com/tzw-wen/kite), were used to filter ProLuCID search results at given FDRs at the spectrum, peptide, and protein levels. Here all controlled FDRs were <1%. The four data sets (two DYRK1A FLAG-IPs and two control FLAG-IPs) were contrasted against their merged data set using Contrast v 1.9 and in-house developed sandmartin v0.0.1. Our in-house developed software, NSAF7 v0.0.1 (10.1021/ac9023999), was used to generate spectral count-based label-free quantification results (distributed normalized spectral abundance factor). QPROT (10.1074/mcp.M800203-MCP200) was used to assess the significant enrichment of affinity-purified proteins compared to negative controls.

### Immunofluorescence

Cells were plated onto pre-coated glass coverslips in a 24-well plate. After 36–48 h of transfection of plasmids as indicated using PEI or Lipofectamine 3000, cells were fixed with 4% paraformaldehyde for 15 min. After being washed three times with PBS, the cells were permeabilized with 0.2% Triton X-100 at RT for 10 min and then washed three times with PBS. Blocking was performed with 5% normal goat serum for 1 h at RT, followed by incubation with primary antibodies at 4 °C overnight. After being washed three times with PBS, the samples were treated with Alexa Fluor® 488-conjugated (A11001, Invitrogen) and Alexa Fluor® 568-conjugated secondary antibodies (A11011, Invitrogen) for 1 h at RT. After being washed with PBS, the samples were treated with DAPI (D3571, Invitrogen) for 5 min at RT and washed with PBS three times. Finally, the coverslips were placed on glass slides and sealed with nail polish. The fluorescence staining was detected with ZEISS 710 confocal laser scanning microscope. Images were processed and analyzed with Zen software.

### In vivo ubiquitination assay

HEK293 cells were transfected with the indicated plasmids for 36–48 h. After rinsing twice with PBS, cells were scraped into 100 μl of lysis buffer (2% SDS, 150 mM NaCl, 20 mM Tris-HCl, pH 8.0), heated for 10 min at 95 °C, diluted with the same lysis buffer lacking SDS to reduce the SDS concentration to 0.2%, and sonicated mildly on ice. Lysates were centrifuged at 17,000 × *g*, 4 °C for 15 min. Then the supernatant was incubated with anti-Flag M2 affinity gel (A2220, Sigma) or incubated with protein A (Millipore, USA) and anti-DYRK1A antibody overnight at 4 °C. Beads were washed three times and boiled with 2× SDS loading buffer for 10 min. Samples were subjected to SDS-PAGE and analyzed by western blotting using the indicated antibodies.

### In vitro ubiquitination assay

*E. coli* BL21 cells were transformed with pGEX-4T-1 or pGEX-4T-1-TRAF2 plasmids, induced by IPTG, and purified by Glutathione Sepharose beads (17-0756-01, GE Healthcare). Flag-DYRK1A was overexpressed in HEK293T cells, cultured for 48 h post transfection, and purified. In vitro ubiquitination assays were performed with the following components: 1 μg purified GST-TRAF2, 2 μg purified Flag-DYRK1A, 100 μM HA-ubiquitin, 100 nM E1, 1 μM E2 enzymes (UbcH5a/b/c and Ubc13/UeV1a (Boston Biochem, Cambridge, MA, USA)), 1 mM ATP in Ubiquitin conjugation reaction buffer. The reaction was run for 3 h at 37 °C. After that, the reaction volume was adjusted to 150 μl, dissociated by heating at 95 °C in 1% SDS(v/v), and diluted 1 : 10 with IP buffer (20 mM Tris-HCl pH 7.4, 150 mM NaCl, 0.5% Triton X-100, 2 mM EDTA, phosphatase and protease inhibitors). The diluted reactions were incubated with anti-Flag M2 affinity gel (A2220, Sigma) overnight at 4 °C. Then beads were washed six times and eluted with 2× SDS loading buffer. Samples were subject to western blotting analysis.

### Statistical analysis

All the statistical data were presented as the means ± SD from at least three independent experiments. The statistical significance of differences between groups were analyzed using the GraphPad Prism software. Differences between individual groups were analyzed using the Student’s *t*-test. A *p*-value < 0.05 was considered significant.

### Mass spectrometry data set accessibility

Mass spectrometry data files are available from Massive at /ftp://massive.ucsd.edu/MSV000085815/ and ProteomeXchange (PXD020533). Original data underlying this manuscript can be accessed from the Stowers Original Data Repository at http://www.stowers.org/research/publications/libpb-1542.

## Results

### DYRK1A interacts with E3 ubiquitin ligase TRAF2

**Fig. 1:**
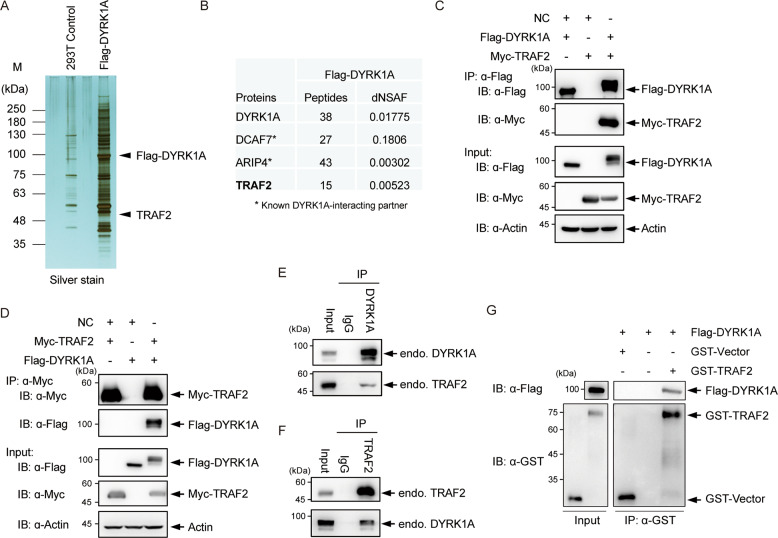
DYRK1A co-purifies with TRAF2. **A** Silver staining of Flag-DYRK1A pull-down eluate. A clonal HEK293T cell line inducibly expressing Flag-DYRK1A [30] was used to generate cytoplasmic extract (S100) using the Dignam protocol. Eluates were analyzed by silver staining and MudPIT analysis. **B** Relative abundance of proteins as analyzed by MudPIT. dNSAF (distributed Normalized Spectral Abundance Factor) values [58] of the identified proteins in the Flag-DYRK1A are shown. **C** Exogenous Flag-DYRK1A interacts with Myc-TRAF2. HEK293 cells were transfected with vector, Flag-DYRK1A, and/or Myc-TRAF2 plasmids and Flag-affinity purified, and blotted with respective antibodies. **D** Exogenous Myc-TRAF2 interacts with Flag-DYRK1A. As in **C**, but Myc-TRAF2 is affinity purified and probed with α-Flag (DYRK1A) antibody. **E** Endogenous DYRK1A interacts with TRAF2. HEK293 cell lysates were immunoprecipitated with normal control rabbit IgG or with α-DYRK1A, and probed with α-DYRK1A and α-TRAF2 antibodies. **F** Endogenous TRAF2 interacts with DYRK1A. HEK293 cell lysates were immunoprecipitated with normal control mouse IgG or α-TRAF2, and probed with α-DYRK1A and α-TRAF2 antibodies. **G** TRAF2 directly interacts with DYRK1A. GST-Vector and GST-TRAF2 were prepared in *E. coli* BL21 cells and Flag-DYRK1A protein was purified from HEK293T cells, respectively. In vitro pull-down assays were performed using GST beads and co-eluted DYRK1A was detected with α-Flag antibody.

We have previously generated stable HEK293T cell lines transformed with inducible Flag-DYRK1A [30, 33]. From these cells, we affinity-purified Flag-DYRK1A from cytoplasmic fractions (S100) and analyzed the eluates by SDS-PAGE and silver staining (Fig. 1A). To identify the interacting proteins, we performed MudPIT analysis and observed enrichment of a number of proteins including previously known interactors including DCAF7, ARIP4 (Fig. 1B) [34–36]. Among the novel interactions of DYRK1A, we found TRAF2, an E3 ubiquitin ligase for both K48- and K63-linked ubiquitination. To confirm the interaction between TRAF2 and DYRK1A, we co-overexpressed Flag-DYRK1A and Myc-TRAF2. To prevent the IgG heavy chain from obscuring the TRAF2 signal, which migrates around 50 kDa in SDS-PAGE, we utilized a secondary antibody recognizing only the light chain. We observed that the overexpressed Flag-DYRK1A pulled down overexpressed Myc-TRAF2 (Fig. 1C). Conversely, overexpressed Myc-TRAF2 pulled down Flag-DYRK1A (Fig. 1D). Interestingly, overexpression of TRAF2 caused the appearance of several new slower migrating bands in the DYRK1A overexpression lane. Further, we co-immunoprecipitated endogenous DYRK1A or TRAF2 from HEK293 whole-cell lysates and found that DYRK1A can pull down TRAF2, and vice versa (Fig. 1E, F). Interestingly, TRAF2 antibody pulled down all the three major isoforms of DYRK1A, as seen by the antibody directed at the C-terminal peptide (Fig. 1F). This suggested that TRAF2 interacts with a domain common to all the three major isoforms of DYRK1A. Further, to perform GST interaction assay, we purified GST-TRAF2 protein from *E. coli* BL21 cells and incubated with Flag-DYRK1A protein purified from transfected HEK293 cells. We observed that GST-TRAF2 efficiently co-eluted with DYRK1A, which indicated a direct interaction between DYRK1A and TRAF2 (Fig. 1G).

### DYRK1A bears a conserved “PVQE” TRAF2-binding site

**Fig. 2:**
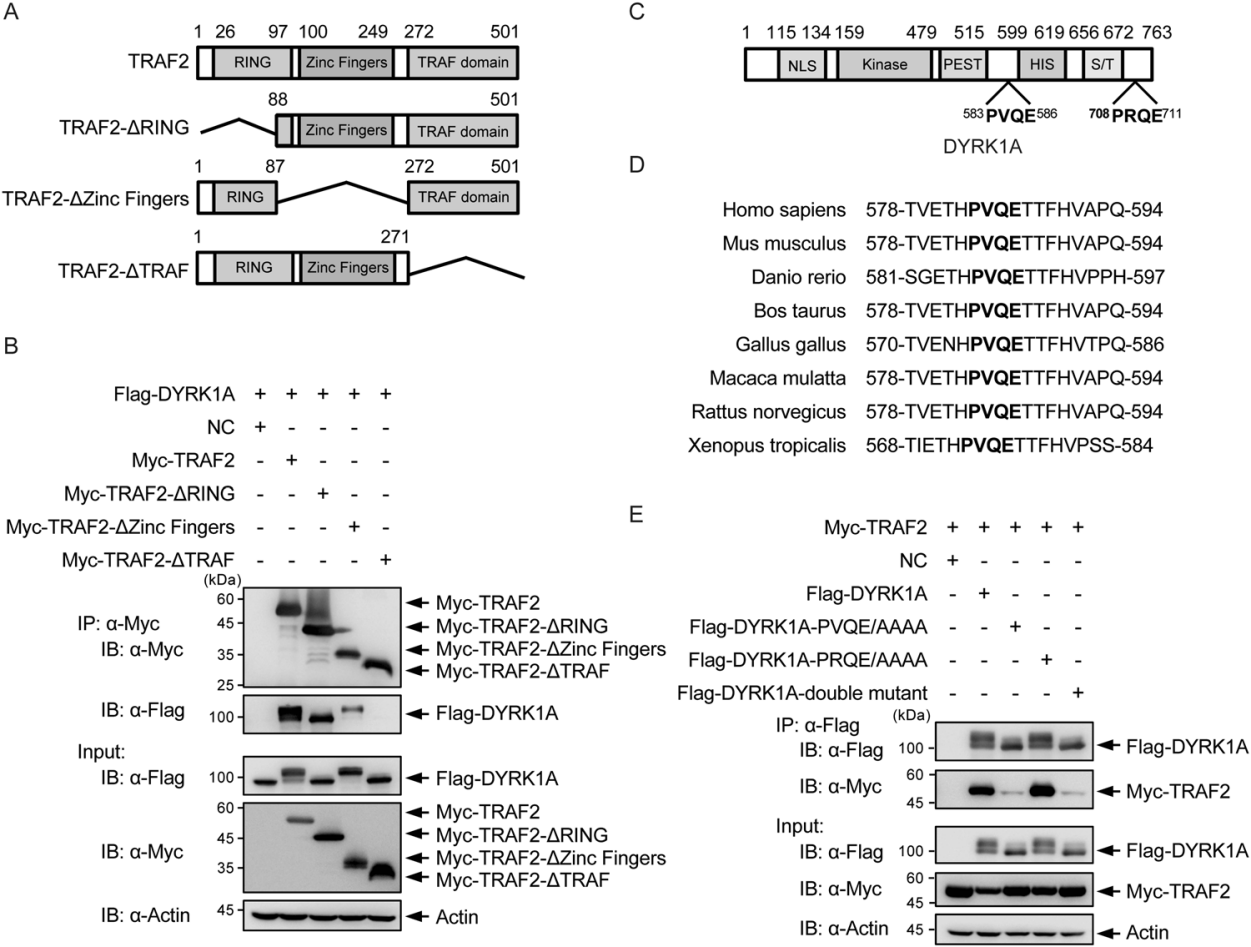
TRAF2 interacts with DYRK1A by binding to TRAF domain-binding motif “PVQE” on DYRK1A. **A** Schematic representation of TRAF2 and its truncations. All constructs are Myc-tagged at N terminus. **B** TRAF2 domains mediating interaction with DYRK1A. Myc-TRAF2 and Myc-TRAF2 deletion constructs were affinity purified from cells co-transfected with Flag-DYRK1A and probed with α-Myc and α-Flag antibodies. All TRAF2 constructs, except ΔTRAF, immunoprecipitated Flag-DYRK1A. **C** Schematic representation of DYRK1A with putative TRAF2-binding motifs marked. **D** Sequence alignment of the major TRAF2-binding motif shows sequence conservation on DYRK1A. Conserved motif is highlighted bold. **E** TRAF2 interaction with PVQE motif on DYRK1A. Flag-DYRK1A, Flag-DYRK1A-PVQE/AAAA, Flag-DYRK1A-PRQE/AAAA, and double mutant were co-expressed with Myc-TRAF2, Flag-affinity purified, and probed with α-Myc antibody. Flag-DYRK1A-PVQE/AAAA and the double mutant have significantly diminished ability to pull down TRAF2.

To analyze the domain/s responsible for interaction between TRAF2 and DYRK1A, we constructed three Myc-TRAF2 truncations as follows: (1) ring deletion (ΔRING), (2) zinc finger domain truncation (ΔZinc Fingers), and (3) TRAF domain truncation (ΔTRAF) (Fig. 2A). Full-length TRAF2, ΔRING, and ΔZinc Fingers but not ΔTRAF interacted with DYRK1A in co-immunoprecipitation experiments (Fig. 2B). In addition, overexpression of ΔZinc Fingers led to the appearance of several slower migrating DYRK1A bands similar to full-length TRAF2 (see input Flag-DYRK1A and Myc-IP Flag-DYRK1A panels). These observations suggested that its TRAF domain is involved in TRAF2 interaction with DYRK1A. Furthermore, E3 ligase activity mediated through RING domain is required for the generation of the slower DYRK1A migrating signals, presumably, ubiquitinated DYRK1A.

TRAF2 recognizes a major ((P/S/A/T)x(Q/E)E) and minor (PxQxxD) motif for binding [37]. DYRK1A protein has two candidate TRAF2-binding motif sites: ^583^PVQE^586^ and ^708^PRQE^711^ (Fig. 2C). A sequence comparison of the potential TRAF2 binding from various organisms, including human, mice, frog, and monkeys, showed that the major sites are conserved in vertebrates (Fig. 2D); however, it is not present in insects or nematodes (data not shown).

We tested the significance of these two sites for interaction and found that DYRK1A PVQE/AAAA and PVQE-PRQE/AAAA-AAAA mutant proteins have significantly lower ability to interact with TRAF2, whereas DYRK1A PRQE/AAAA mutation alone does not alter the interaction significantly compared to wild-type (WT) DYRK1A (Fig. 2E). Moreover, TRAF2 overexpression did not cause the appearance of slower migrating bands in DYRK1A PVQE/AAAA mutant protein and in DYRK1A PVQE-PRQE/AAAA-AAAA mutant protein. Taken together, the above experiments suggest that TRAF2 interacts with DYRK1A through its TRAF domain and binds to its binding motif PVQE on DYRK1A.

### TRAF2 mediates K63-linked ubiquitination of DYRK1A

**Fig. 3:**
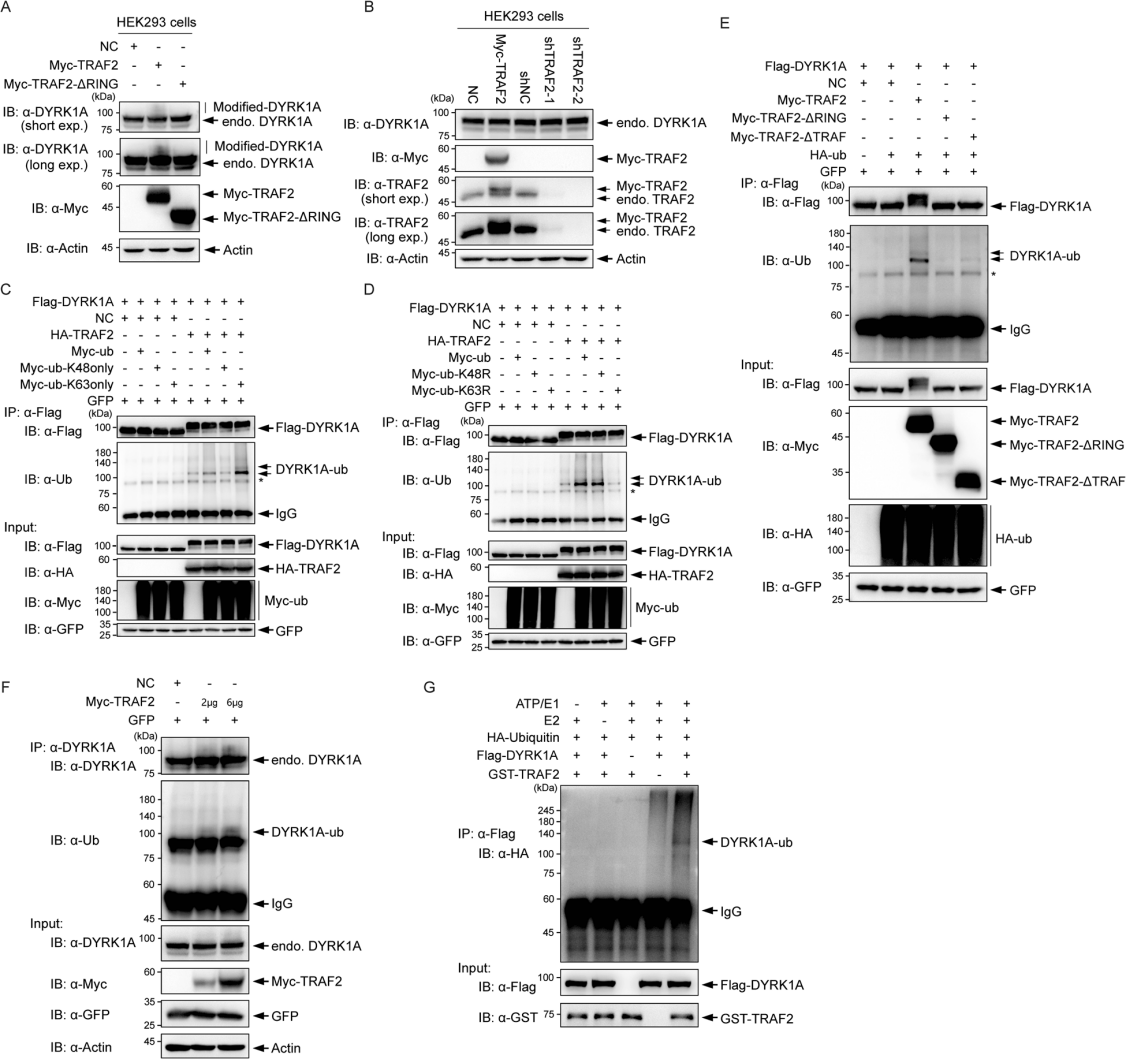
TRAF2 mediates K63-linked ubiquitination of DYRK1A. **A** The effect of overexpression of TRAF2 or its RING mutant on retarded migration of DYRK1A using SDS-PAGE. **B** Analysis of DYRK1A protein levels after overexpression or knockdown of TRAF2 in HEK293 cells. **C** In vivo ubiquitination assay using Myc-Ub with all, except K48 or K63, lysines mutated. Flag-DYRK1A was affinity purified from HEK293 cells co-transfected with Flag-DYRK1A, and/or HA-TRAF2, Myc-Ub (WT, K48-only, K63-only), and GFP as indicated for 36–48 h. Lysates and immunoprecipitates were immunoblotted with α-Myc, α-Flag, α-HA, and α-Ubiquitin antibodies. **D** In vivo ubiquitination assay using Myc-Ub with K48R or K63R mutation. As in **C**, Flag-DYRK1A was affinity purified from HEK293 cells co-transfected with Flag-DYRK1A, and/or HA-TRAF2 and GFP, but not Myc-Ub-K48R or Myc-Ub-K63R, were used. The DYRK1A-Ub signal was similar to Myc-Ub-WT in Myc-Ub-K48R, but absent in Myc-Ub-K63R. **E** Analysis of TRAF2 domains responsible for ubiquitination of DYRK1A. Flag-DYRK1A was affinity purified from HEK293 cells co-transfected with Flag-DYRK1A and/or Myc-TRAF2, Myc-TRAF2 truncates, HA-Ub, and GFP as indicated for 36–48 h. Immunoprecipitates were probed with α-Ubiquitin antibody. **F** Analysis of ubiquitination status of endogenous DYRK1A with increasing expression of TRAF2. Endogenous DYRK1A was affinity purified from the cells co-transfected with GFP and increasing amounts of Myc-TRAF2 as indicated for 36–48 h. Lysates and immunoprecipitates were immunoblotted with α-Myc, α-DYRK1A, and α-Ubiquitin antibodies. **G** TRAF2 was able to ubiquitinate DYRK1A in vitro. Flag-DYRK1A proteins were incubated with adenosine triphosphate, HA-Ub, E1, and E2, along with GST-TRAF2 for in vitro ubiquitination of DYRK1A.

We had observed that overexpression of TRAF2 did not significantly affect the protein level of overexpressed DYRK1A and endogenous DYRK1A but caused retarded migration of DYRK1A on SDS-PAGE (Figs. 1C Input, 1D Input, 2B Input, and 3A). These additional signals were abolished in E3 Ligase activity-deficient ΔRING TRAF2 mutant (Figs. 2B Input and 3A). We analyzed if overexpression or knockdown of TRAF2 affected bulk levels of endogenous DYRK1A protein and found no significant changes (Fig. 3B). TRAF2 mediates K63-linked or K48-linked ubiquitination of its substrates [20–23, 38, 39]. We therefore assessed whether DYRK1A could be ubiquitinated by TRAF2 and what is the nature of its linkage. To determine the nature of the linkage, we first co-transfected WT Myc-tagged Ub (WT Myc-Ub) or Myc-Ub, which has all lysines mutated to alanine, except K48 (K48-only Myc-Ub), or Myc-Ub, which has all lysines mutated to alanine, except K63 (K63-only Myc-Ub) along with Flag-DYRK1A and/or HA-TRAF2, and performed Flag affinity purification to enrich DYRK1A and analyze its ubiquitinated forms. Overexpression of “K63-only Myc-Ub,” but not “K48-only Myc-Ub,” lead to the appearance of ~120 kDa signal. Moreover, the signal intensity increased when TRAF2 was co-expressed along with WT Myc-Ub (Fig. 3C lane 6), and increased further, with “K63-only Myc-Ub” (Fig. 3C lane 8). This suggested that a fraction of cellular DYRK1A could be K63 ubiquitinated and TRAF2 can mediate this modification. Further, we co-expressed WT Myc-Ub, K48R Myc-Ub, or K63R Myc-Ub along with Flag-DYRK1A and/or HA-TRAF2. Enriched Flag-DYRK1A showed strong ubiquitin signal in the presence of WT Myc-Ub and K48R Myc-Ub (Fig. 3D lanes 6 and 7), but not in K63R Myc-Ub (Fig. 3D lane 8), and thus we verified that the ~120 kDa signal is indeed K63-ubiquitinated DYRK1A. Interestingly, we found that TRAF2 ΔRING mutant completely abrogated DYRK1A ubiquitination (Fig. 3E lane 4) and TRAF2 ΔTRAF mutant (Fig. 3E lane 5) had significantly lower ability to ubiquitinate DYRK1A compared to WT TRAF2 (Fig. 3E lane 3). Moreover, increasing amounts of TRAF2 expression caused increased ubiquitination level of endogenous DYRK1A in a dose-dependent manner (Fig. 3F). To further confirm whether TRAF2 is a direct ubiquitin E3 ligase for DYRK1A, we performed in vitro ubiquitination assay in the presence of recombinant human GST-TRAF2 and purified Flag-DYRK1A. Remarkably, TRAF2 was able to ubiquitinate DYRK1A in vitro (Fig. 3G). Taken together, these results suggest that TRAF2 promotes K63-linked ubiquitination of a partial pool of cellular DYRK1A.

### TRAF2 is critical for translocation of DYRK1A to a detergent-insoluble fraction

**Fig. 4:**
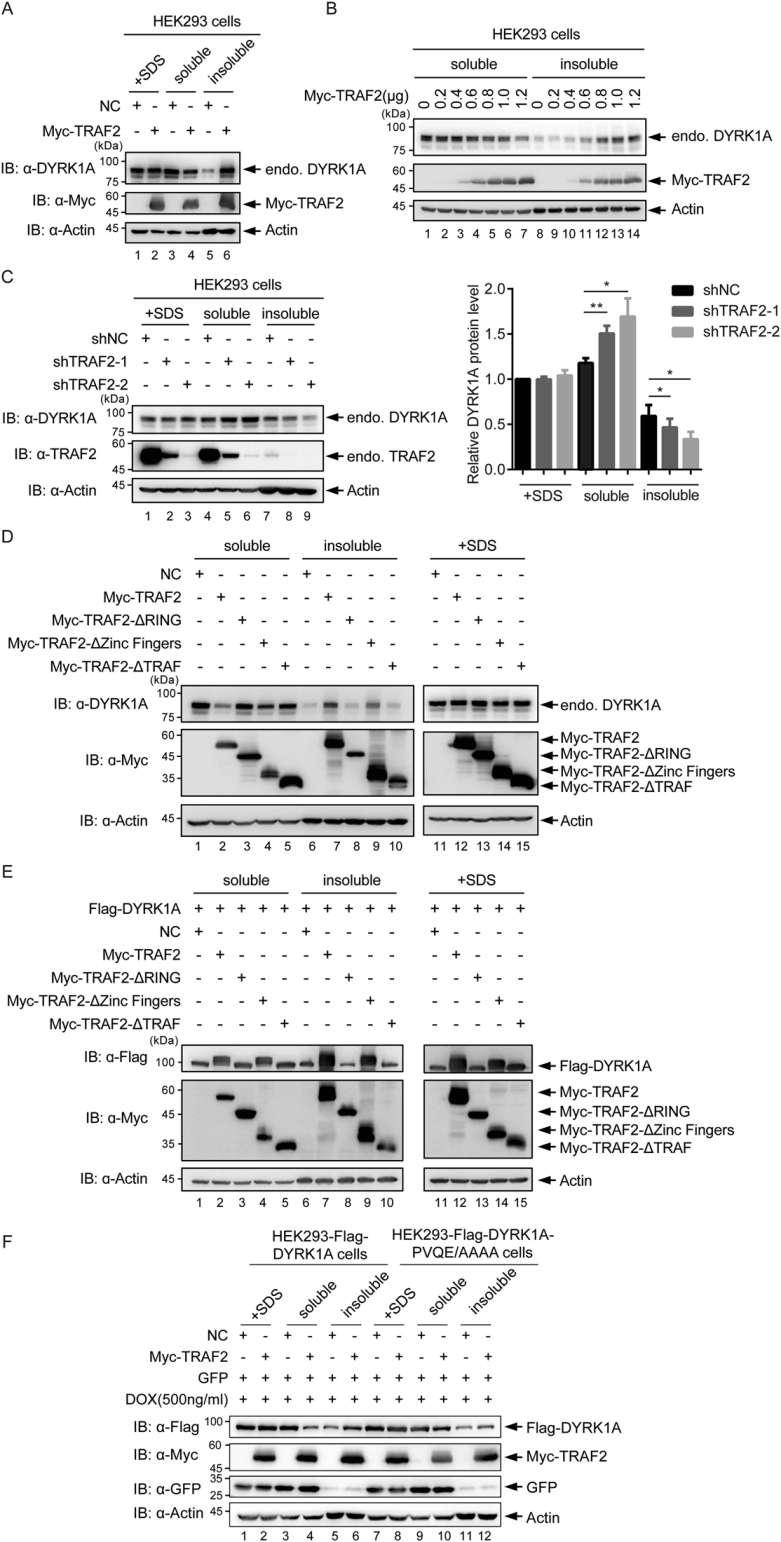
TRAF2 is critical for translocation of DYRK1A to a detergent-insoluble fraction. **A** Distribution of DYRK1A in soluble and insoluble fractions. HEK293 cells transfected with Myc-TRAF2 were lysed in Tris buffer containing 0.5% Triton X-100, and soluble and insoluble fractions were separated by centrifugation. Whole-cell lysates were prepared by directly lysing the cells in SDS loading buffer. **B** DYRK1A distribution in soluble and insoluble fractions with increasing expression of TRAF2. Increasing amounts of Myc-TRAF2 were transfected and cells fractionated to obtain soluble and insoluble fractions as in **A**. With increasing amounts of TRAF2, soluble DYRK1A decreases, whereas the insoluble fraction increases. **C** Knockdown of TRAF2 affects distribution of DYRK1A in soluble and insoluble fractions. TRAF2 was silenced using two different shRNAs and cells fractionated. Amount of DYRK1A in the soluble fraction increases upon TRAF2 knockdown, whereas it decreases in the insoluble fraction. Also, the effect depends on the efficiency of TRAF2 silencing. Right panel shows quantification of three independent experiments. Data were shown as mean ± SD, *P < 0.05 and **P < 0.01. **D** Analysis of TRAF2 domains responsible for distribution of DYRK1A to soluble and insoluble fractions. TRAF2 truncates and full-length TRAF2 were transfected in HEK293 cells, and cells fractionated. The presence of endogenous DYRK1A in soluble and insoluble fractions were determined. RING domain and TRAF domain truncates were unable to translocate DYRK1A to insoluble fraction. **E** Analysis of TRAF2 domains responsible for distribution of exogenous DYRK1A to soluble and insoluble fractions. Exogenous Flag-DYRK1A was transfected additionally to the **D** experiment and distribution of Flag-DYRK1A probed. **F** Analysis of distribution of Flag-DYRK1A-PVQE/AAAA in soluble and insoluble fractions. Lentiviruses were used to express Flag-DYRK1A or Flag-DYRK1A-PVQE/AAAA in HEK293 cells and their distribution analyzed after overexpression of TRAF2. Flag-DYRK1A-PVQE/AAAA does not translocate to insoluble fraction as efficiently as WT DYRK1A.

K63-linked ubiquitination has been linked to a number of functions including signal transduction, protein trafficking, protein–protein interaction, and DNA damage response. TRAF2-mediated K63-linked ubiquitination has been to shown to function in protein–protein interaction and enzymatic activity [20–23]. To gain insight into the potential effects of TRAF2 on DYRK1A function, and if TRAF2 promotes translocation of DYRK1A to endocytic vesicles or endosomes, we generated Triton X-100 (0.5%)-soluble and -insoluble fractions. The soluble fraction contains the majority of soluble cytoplasmic and nuclear proteins, whereas cytoskeletal and vesicles remain in the insoluble fraction. A fraction of TRAF2 localizes to the Triton X-100-insoluble fraction [40–42]. Further, it has been reported that a cellular pool of DYRK1A is located in Triton X-100-resistant manner [12] and interacts with a number of vesicular proteins, including Sprouty 2, Synaptojannin 1, Amphiphysin, Dynamin 1, Endophilin 1, and phosphorylates several of them [12, 13, 43, 44]. After TRAF2 overexpression, we found that a significant amount of endogenous DYRK1A became sequestered into the insoluble fraction (Fig. 4A). Further, increasing amounts of TRAF2 expression caused increased migration of endogenous DYRK1A to detergent-insoluble fraction in a dose-dependent manner (Fig. 4B).

To further understand the dependency of this phenomenon on TRAF2 dosage and to avoid drawing conclusions based mainly on overexpression experiments, we analyzed localization of endogenous DYRK1A after reducing TRAF2 by shRNA in multiple cell lines. Knockdown of TRAF2 in HEK293 cells with two different shRNAs consistently increased the DYRK1A protein in the soluble fraction (Fig. 4C). Similar results were obtained in SH-SY5Y cells (data not shown). These observations suggest that TRAF2 is critical for translocation of DYRK1A into membranous vesicles and is probably a common phenomenon in various tissues.

To dissect the domain of TRAF2 responsible for DYRK1A translocation, we analyzed the effects of overexpressing TRAF2 truncations in HEK293 cells. DYRK1A was translocated to detergent-insoluble fraction when TRAF2-WT or the ΔZinc Fingers were overexpressed. In contrast, the ΔRING or ΔTRAF mutants failed to induce translocation of endogenous DYRK1A to insoluble fraction (Fig. 4D). We also analyzed whether overexpressed DYRK1A behaved in a similar manner upon overexpression of TRAF2 truncations and found a similar distribution of DYRK1A (Fig. 4E). Further, we asked whether the TRAF2-binding motif PVQE sequence present on DYRK1A protein is required for translocation of DYRK1A to detergent-insoluble fraction. We used an inducible lentiviral vector to express DYRK1A WT or PVQE/AAAA mutant in HEK293 cells and observed that upon TRAF2 overexpression, PVQE/AAAA mutant remained mostly soluble compared to WT DYRK1A (Fig. 4F, compare lanes 4 and 6 with lanes 10 and 12). These findings suggest that TRAF2 causes translocation of DYRK1A to a detergent-insoluble fraction, likely by ubiquitinating it.

### TRAF2 promotes phosphorylation of SPRY2 by DYRK1A

Translocation of ubiquitinated DYRK1A into vesicles, where it can potentially phosphorylate multiple proteins, suggests that DYRK1A might have a widespread role in vesicle biology. DYRK1A has been shown to regulate synaptic vesicle endocytosis and recycling in *Drosophila* as well as in mammalian system [11, 43]. DYRK1A phosphorylates a number of proteins in the vesicles, including SPRY2, Synaptojanin 1, Endophilin 1, and Dynamin 1. To understand the direct impact of ubiquitinated DYRK1A in the vesicles, we briefly scanned some of the known DYRK1A substrates in vesicles, including SPRY2, Synaptojanin 1, Dynamin 1, and α-synuclein for an increase in phosphorylation; however, due to absence of readily available antibodies specific to phosphorylated forms of these proteins, we are unable to build a detailed map of substrates of ubiquitinated DYRK1A. We chose to further investigate SPRY2 and determine whether it is one of the substrates of ubiquitinated DYRK1A.

**Fig. 5:**
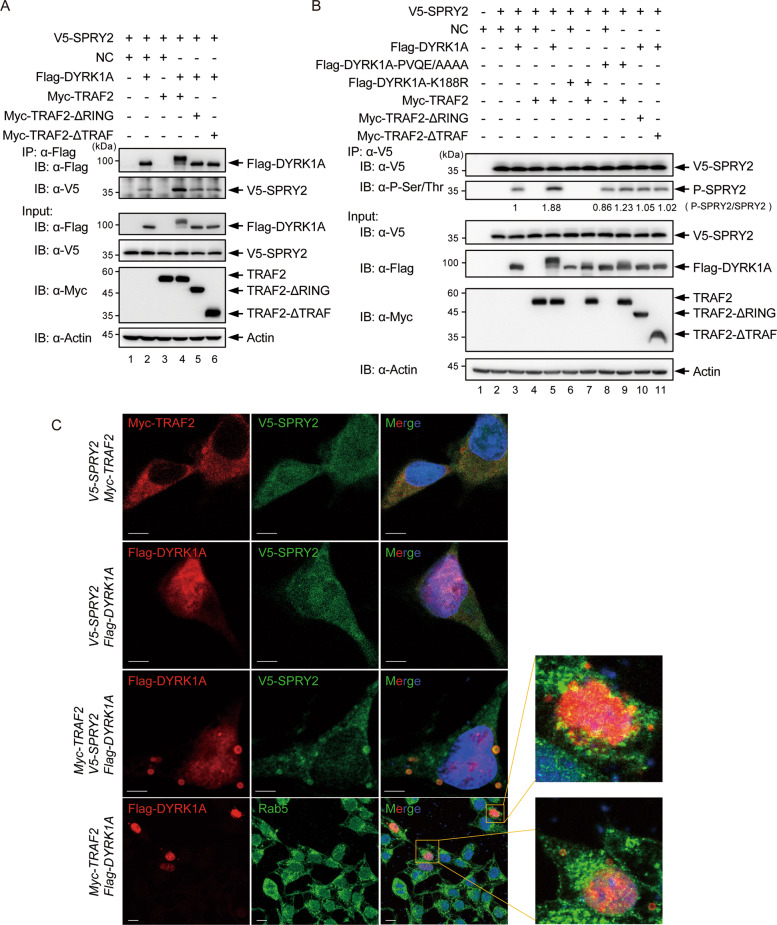
TRAF2 promotes DYRK1A-mediated phosphorylation of SPRY2. **A** Interaction analysis between DYRK1A and SPRY2 in the presence of WT TRAF2 or its truncates. V5-SPRY2 was co-transfected with Flag-DYRK1A, Myc-TRAF2, or its different truncations into HEK293T cells and Flag-affinity purified. Immunoprecipitates were probed with α-Flag and α-V5 to analyze the enrichment of SPRY2 in DYRK1A pulldowns after overexpression of TRAF2 and truncates. **B** Analysis of phosphorylation status of SPRY2 in the presence of DYRK1A and TRAF2. V5-SPRY2 was co-transfected with Flag-DYRK1A (WT, PVQE/AAAA or K188R) and/or Myc-TRAF2 (WT, ΔRING or ΔTRAF) in HEK293T cells, followed by V5-affinity purification. Immunoprecipitates were probed with α-phospho-pan-Ser/Thr antibody. Full-length TRAF2, but not ΔRING or ΔTRAF deletions, was able to promote phosphorylation of SPRY2 in the presence of wild-type DYRK1A. Also, Flag-DYRK1A-PVQE/AAAA mutant did not promote the phosphorylation of SPRY2. **C** Cellular localization of V5-SPRY2, Myc-TRAF2, Flag-DYRK1A, and Rab5. HEK293T cells were co-transfected with V5-SPRY2, Flag-DYRK1A, and/or Myc-TRAF2, and immunostained with respective antibodies. Images were acquired using ZEISS 710 confocal laser scanning microscope. The first three panels, scale bar = 25 μm. The last panel, scale bar = 1 μm.

DYRK1A has been shown to phosphorylate SPRY2 on Thr75, and possibly other sites [45, 46], which in turn prevents endocytic degradation of EGFR [16, 17]. We first asked whether TRAF2 can promote the interaction of DYRK1A with SPRY2. We co-expressed V5-SPRY2, Flag-DYRK1A, and/or MYC-TRAF2 (WT, ΔRING, and ΔTRAF) in HEK293T cells and affinity-purified Flag-DYRK1A, then analyzed the enrichment of SPRY2. We observed that V5-SPRY2 was more enriched in Flag-DYRK1A eluates when co-transfected with WT TRAF2 (lane 4), compared to empty vector (lane 2), ΔRing (lane 5), and ΔTRAF (lane 6) mutants (Fig. 5A), suggesting that the translocation of ubiquitinated DYRK1A into vesicles enabled increased interaction between DYRK1A and SPRY2. We further tested whether phosphorylation of SPRY2 could be promoted by TRAF2. We co-transfected V5-SPRY2 along with various constructs of DYRK1A and TRAF2, affinity-purified V5-SPRY2, and analyzed its phosphorylation status. We found that WT TRAF2, but not RING domain deletion or TRAF domain deletion, increased the phosphorylation level of Sprouty 2 (Fig. 5B lanes 5, 10, and 11). Flag-DYRK1A-PVQE/AAAA mutant also did not promote the phosphorylation of SPRY2 (Fig. 5B lanes 8 and 9). The catalytically inactive Flag-DYRK1A (K188R mutant) could not phosphorylate SPRY2 with/without TRAF2 overexpression (Fig. 5B lanes 6 and 7).

To further analyze the cellular colocalization of DYRK1A, TRAF2, and SPRY2, we performed confocal microscopy in HEK293T cells. TRAF2 localizes within a number of cellular structures, including the cytoplasm, lipid rafts in the plasma membrane [47], mitochondria [48, 49], and endoplasmic reticulum [42]. Overexpressed TRAF2 also localizes to the vesicles, in both mouse and human cells [50]. As reported previously, SPRY2 showed a diffused cytoplasmic staining (Fig. 5C) [51], whereas overexpressed Flag-DYRK1A showed strong nuclear staining and weak cytoplasmic staining (Fig. 5C) [52]. Upon co-expression of V5-SPRY2, Myc-TRAF2, and Flag-DYRK1A, we observed colocalization of V5-SPRY2 and DYRK1A in the vesicles (Fig. 5C). Further, we found that some of the vesicles can colocalize with endosome marker Rab5 (Fig. 5C). These experiments suggest that TRAF2 can promote the interaction of DYRK1A with SPRY2 and thus promote the phosphorylation of SPRY2 by DYRK1A.

### TRAF2 regulates EGFR protein stability through DYRK1A

**Fig. 6:**
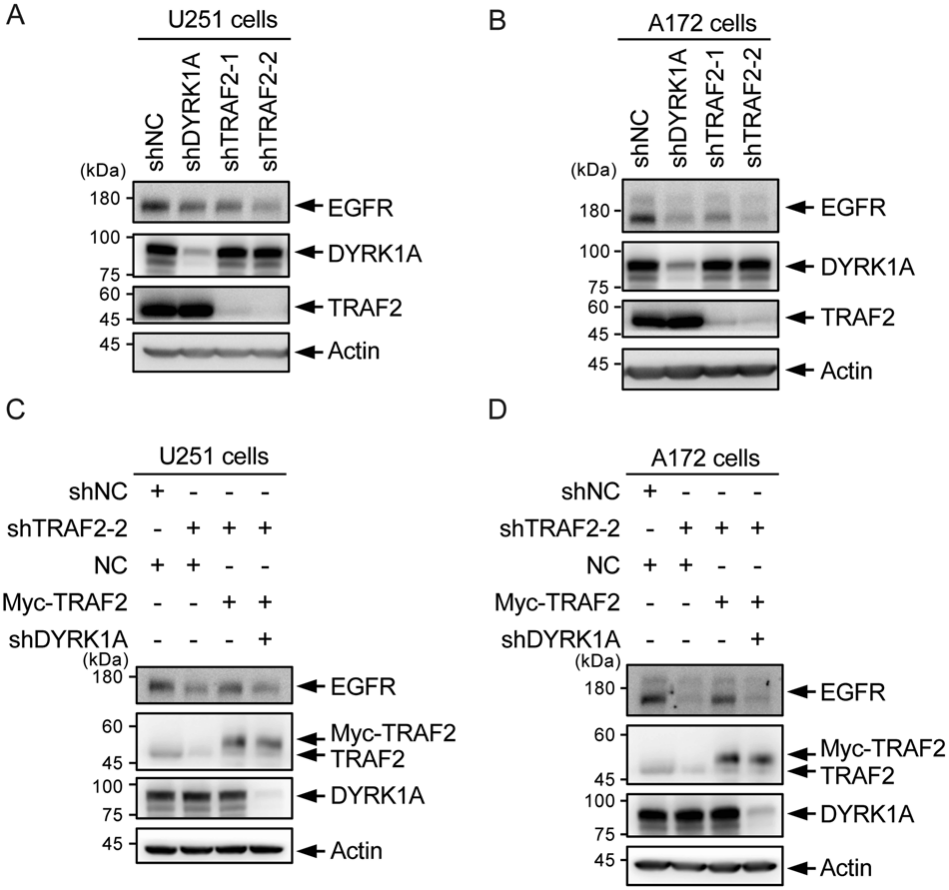
TRAF2 regulates EGFR protein stability through DYRK1A. **A** TRAF2 knockdown affects EGFR protein level. DYRK1A and TRAF2 were silenced by shRNAs in U251 cells and cell lysates probed with EGFR, DYRK1A, and TRAF2 antibodies. **B** EGFR protein levels were analyzed in A172 cells after shRNA-mediated knockdown as in **A**. **C** DYRK1A knockdown causes that TRAF2 overexpression does not rescue EGFR protein level in TRAF2-silenced cells. U251 cells, silenced alone with TRAF2 shRNA or co-silenced with TRAF2 and DYRK1A shRNA were transfected with Myc-TRAF2, and protein level of EGFR analyzed. **D** Same as in **C** but A172 cell line was used.

SPRY2 regulates EGFR stability, which is partly dependent on DYRK1A through its regulation of SPRY2 [17, 53, 54]. Inhibition of DYRK1A in glioma cells destabilizes EGFR and reduces EGFR-dependent glioblastoma growth [17]. To test whether TRAF2 can regulate the stability of EGFR in glioma cells, we performed knockdown of DYRK1A and TRAF2 in human glioma cell lines U251 and A172, and analyzed the protein level of EGFR. As previously reported [16, 17], knockdown of DYRK1A resulted in a reduction of EGFR protein level. Similarly, knockdown of TRAF2, with two different shRNAs, also leads to a reduction of EGFR levels (Fig. 6A, B). Further, we observed that the overexpression of TRAF2 cannot rescue the reduced EGFR levels caused by DYRK1A knockdown in U251 and A172 cells, suggesting that TRAF2-facilitated EGFR protein stabilization is mediated through DYRK1A, and probably through other unknown mechanism (Fig. 6C, D). Taken together, our data suggests that TRAF2–DYRK1A–SPRY2 interaction is important for regulating EGFR stability in glioma cells.

### Depletion of DYRK1A inhibits the growth of glioma cells mediated by TRAF2

**Fig. 7:**
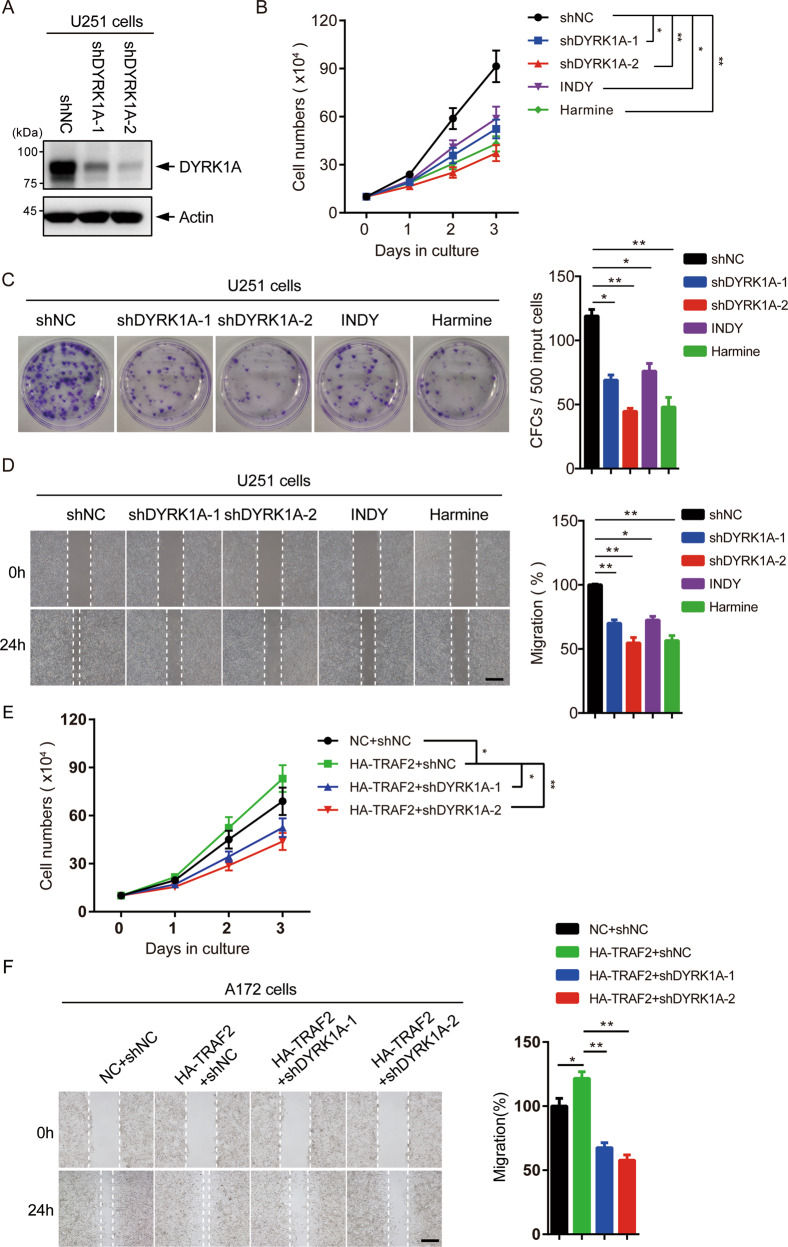
Depletion of DYRK1A inhibits the growth of glioma cells mediated by TRAF2. **A** U251 cells were infected with lentivirus-expressing shRNAs targeting control or DYRK1A. After selection with puromycin for 5 days, cells were lysed and level of DYRK1A analyzed by western blotting. **B** U251 cells were incubated with 15 μM INDY or 15 μM Harmine, or were infected with shNC or shDYRK1A lentivirus and selected with puromycin. Cell proliferation of the treated U251 cells was measured (*n* = 3 independent experiments). Data were shown as mean ± SD, *P < 0.05 and **P < 0.01. **C** Colony-forming capacities of U251 cells treated as in **B** were measured (CFC, colony-forming cells). Representative images were shown (left) and the quantification of three independent experiments was plotted (right). Data were shown as mean ± SD, *P < 0.05 and **P < 0.01. **D** The wound-healing assay was used to analyze the migration ability of U251 cells treated as in **B**. Left panel, representative images were shown. Right panel, the quantification of three independent experiments was plotted. Data were shown as mean ± SD, *P < 0.05 and **P < 0.01, scale bar = 500 μm. **E** A172 cells were infected with lentivirus-expressing control, HA-TRAF2 and/or DYRK1A shRNAs. Cell proliferation of the A172 cells was measured (n = 3 independent experiments). Data were shown as mean ± SD, *P < 0.05 and **P < 0.01. **F** The wound-healing assay was used to analyze the migration ability of the treated A172 cells treated as in **E**. Representative images were shown (left) and the quantification of three independent experiments was plotted (right). Data were shown as mean ± SD, *P < 0.05 and **P < 0.01, scale bar = 500 μm.

Recent reports show that TRAF2 is of prognostic significance in glioblastoma and its inhibition suppresses proliferation of glioblastoma cells [28, 29]. To examine whether TRAF2-promoted proliferation of glioblastoma cells is mediated through DYRK1A, we first performed shRNA-mediated knockdown of DYRK1A using lentiviruses (Fig. 7A). We also inhibited DYRK1A kinase activity using two different DYRK1A inhibitors INDY or Harmine (Fig. 7B). Both the treatments significantly suppressed the growth and colony-forming capacities of U251 cells (Fig. 7B, C). Also, DYRK1A inhibition resulted in a significant reduction in the rate of cell migration compared to the control (Fig. 7D). As has been previously suggested [17], our results also show that DYRK1A inhibition suppresses the growth and migration of glioma cells. We then overexpressed TRAF2 in DYRK1A-silenced A172 cells and observed that TRAF2 cannot rescue the growth inhibition caused by DYRK1A knockdown (Fig. 7E, F). These results imply that DYRK1A is an important downstream target of TRAF2 in glioma cells.

## Discussion

Understandings of DYRK1A functions has steadily increased in the past two decades and we currently know more than 20 substrates that can be phosphorylated by DYRK1A in various cellular compartments. DYRK1A is a highly conserved protein in vertebrates and contains a kinase domain, a DYRK1A homology (DH) domain, two nuclear localization signals, a PEST region, a histidine-rich domain, and C-terminal region rich in serine and threonine [44]. These domains may interact with proteins that cause translocation of DYRK1A to various sites within a cell. Whether these domains directly or indirectly regulate the kinase activity of DYRK1A is a very important question. Earlier studies demonstrated that DYRK1A is auto-activated by autophosphorylation of the critical activation-loop tyrosine during translation [55], and that the phosphorylation of S520 could promote the kinase activity further by allowing the binding of 14-3-3β [56]. A more recent study of the structure of DYRK family of kinases has implicated the DH domain and NAPA region in its kinase activity [57]. Therefore, it is likely that auto-activation allows DYRK1A to phosphorylate some or many substrates within the cell, whereas interaction with other proteins may either promote its interaction with other substrates or regulate its translocation to subcellular compartments [56]. Therefore, an important mode of regulation of kinase activity of DYRK1A could be compartmentalization. DYRK1A has been shown to be localized in many different cellular organelles, where it may phosphorylate its substrates. In vesicles, DYRK1A phosphorylates SPRY2, Synaptojanin 1, Dynamin 1, and probably other proteins.

In this study we show that DYRK1A is posttranslationally modified by TRAF2, an important adaptor molecule and an E3 ligase of the TNFα receptor superfamily. Our data suggests that TRAF2 interacts with a partial pool of cytoplasmic DYRK1A, binds to its “PVQE”-binding motif, and performs its K63-linked ubiquitination (Fig. 3C, D). Using the PVQE-binding site mutant and TRAF2 RING domain mutant, we demonstrated that K63-linked ubiquitination is key for the translocation of DYRK1A to the vesicles, present in the Triton X-100-insoluble fractions (Fig. 4D, F). To analyze whether this translocation is a general phenomenon in various cellular contexts, and if it occurs endogenously, we have analyzed the translocation of DYRK1A after knocking down TRAF2 in two different cell lines. Both cells showed increased translocation of DYRK1A from insoluble to soluble fraction. This significant change in the DYRK1A localization after TRAF2 knockdown suggests that a sizable fraction of DYRK1A is ubiquitinated and translocated to the endosome, and possibly to other membrane-bound fractions.

**Fig. 8:**
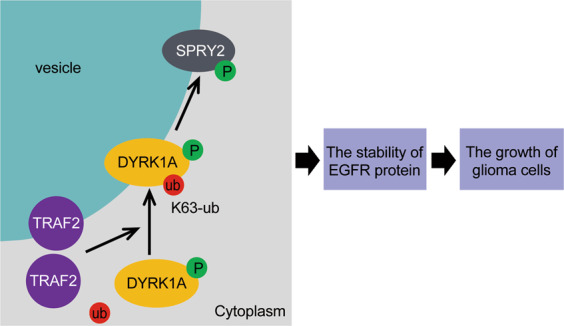
Working model of TRAF2–DYRK1A–SPRY2 regulation of EGFR and glioma cell growth. TRAF2 mediates K63-linked ubiquitination of DYRK1A, which leads to its translocation to vesicles, where it interacts with and phosphorylates SPRY2. Phosphorylated SPRY2 negatively regulates the endocytosis and recycling of EGFR, which promotes the growth of glioma cells.

Our study provides a mechanistic understanding of how DYRK1A is translocated to these vesicular structures and regulates processes mediated through vesicles and the endocytotic system through TRAF2 (Fig. 8). Although TRAF2 is very well-studied, its function in signaling pathways other than the TNFα pathway is barely understood. Recent studies have begun to unravel the role of TRAF2 in cancers, including in the glioma [29]. To the best of our knowledge, regulation of EGFR stability by TRAF2 has not yet been reported. It is interesting to know that there could be a link between TRAF2, an important adaptor molecule of the TNFα pathway and EGFR. It raises some important questions, including if TNFα pathway can regulate the EGFR pathway and in what contexts. Also, it would be interesting to know whether activation of the non-canonical NF-κB pathway by CD40, BAFF receptor, lymphotoxin-β-receptor, or other inducers, which lead to degradation of TRAF2 [19], affect the ubiquitination status and localization of DYRK1A, and if the TRAF2–DYRK1A–SPRY2 axis can mediate stabilization of EGFR and activation of the EGFR-signaling pathway. The addition of TRAF2 to the DYRK1A–SPRY2–EGFR axis has broadened the complexity of regulation of EGFR and its dynamics in cancer signaling.
